# Dendritic Cells Transfected with scFv from Mab 7.B12 Mimicking Original Antigen gp43 Induces Protection against Experimental Paracoccidioidomycosis

**DOI:** 10.1371/journal.pone.0015935

**Published:** 2011-01-07

**Authors:** Karen S. Ferreira, Andrea Q. Maranhão, Maria C. C. Garcia, Marcelo M. Brígido, Suelen S. Santos, José D. Lopes, Sandro R. Almeida

**Affiliations:** 1 Departamento de Ciências Biológicas do Instituto de Ciências Ambientais, Químicas e Farmacêuticas da Universidade Federal de São Paulo, São Paulo, Brazil; 2 Departamento de Biologia Celular da Universidade de Brasília, Brasília, Brazil; 3 Departamento de Análises Clínicas e Toxicológicas da Universidade de São Paulo, São Paulo, Brazil; 4 Departamento de Microbiologia, Imunologia e Parasitologia da Universidade Federal de São Paulo, São Paulo, Brazil; University of Minnesota, United States of America

## Abstract

Paracoccidioidomycosis (PCM), endemic in Latin America, is a progressive systemic mycosis caused by *Paracoccidioides brasiliensis (P. brasiliensis)*, which primarily attacks lung tissue. Dendritic cells (DCs) are able to initiate a response in naïve T cells, and they also participate in Th-cell education. Furthermore, these cells have been used for therapy in several disease models. Here we transfected DCs with a plasmid (pMAC/PS-scFv) encoding a single chain variable fragment (scFv) of an anti-Id antibody that is capable of mimicking gp43, the main antigenic component of *P. brasiliensis*. First, Balb/c mice were immunized subcutaneously with pMAC/PS-scFv and, after seven days, scFv protein was presented to the regional lymph nodes cells. Moreover, we showed that the DCs transfected with scFv were capable of efficiently activating proliferation of total lymph node cells and inducing a decrease in lung infection. Therefore, our results suggested that the use of scFv-transfected DCs may be a promising therapy in the paracoccidioidomycosis (PCM) model.

## Introduction

Paracoccidioidomycosis (PCM) is a mycotic disease caused by a terminally dimorphic fungus *Paracoccidioides brasiliensis (P. brasiliensis)*, which initiates a deep mycosis and a primary attack on the lung tissue. Epidemiological and experimental evidence suggests that natural infection is initiated after inhalation of the conidia produced by the mycelial form of the fungus [1].

The main antigenic component of the *P. brasiliensis* is a 43-kDa glycoprotein that has been shown to elicit strong antibody and cellular immune responses in human and experimental models [2], [3]. Antibodies against this glycoprotein can be detected during all stages of the disease. The analysis of the isotype representation of the IgG reactivity indicated that the two forms of the disease (sub-acute and chronic) have distinct profiles. IgG4 reactivity was associated with the sub-acute form and IgG2 was only seen in chronic form patients [4]. Based on the knowledge that a conversion to IgG4 is dependent on interleukin-4 and that the conversion to IgG2 is influenced by interferon-γ [5], [6], it is suggested that the sub-acute form is related to a Th2-type pattern of immune response, while a chronic form is related to a protective response (Th1-type) in the PCM disease.

According to the network hypothesis proposed by Jerne et al [7], anti-idiotypic (anti-Id) antibodies are a component of the normal immune response, which results in a web of interacting idiotypic (Id) antibodies. Id are the sum of idiotopes or serologically determined antigenic determinants unique to an antibody or group of antibodies [8], [9]. Anti-Id antibodies recognize antigenic determinants that overlap in the combining site that is in contact with the original antigen; thus, they are supposed to carry its “internal image”. Although those anti-Id antibodies, also known as Ab2-β, are able to mimic the antigen, they represent a small fraction of all anti-Id antibodies produced [10], [11]. Previous research showed that mice immunized with anti-gp43 monoclonal antibodies (Mabs) (Ab1) unleashed the idiotypic cascade and produced both anti-Id antibodies (Ab2) as well as anti-anti-Id antibodies (Ab3). Ab2 Mabs named 7.B12 inhibited (>95%) the binding of gp43 to Mab 17C (Ab1), suggesting that this anti-Id Mab bound to the idiotope, thus fulfilling the “internal image” criteria proposed by Nisonoff and Lamoyi [12]. To elucidate whether the Ab2-β Mab (7.B12), rather than gp43, may act as an antigen in serological assays, the sera from PCM patients were tested. An ELISA test using Ab2-β bound to the solid phase allowed for the serological monitoring of patients after antifungal therapy, and the test produced an equivalent curve when compared with an ELISA that employed purified gp43. We also observed a T-cell proliferation response when mice were immunized with Ab2-β and when their cells were exposed to gp43 *in vitro* [13]. Previous studies showed that anti-Id are superior to peptides or antigens in inducing viral epitopes to CD4^+^ T cells activation by APCs [14]. Therefore, we suggested that Ab2-β 7.B12, which contains a T-cell epitope, may be used in a protection assay.

Antibody based therapy has gained increased acceptance with several Mabs currently used as therapeutic agents or in late stage clinical trials [15]. However, Mabs of either human or mouse origin may remain in the circulation and can generate unwanted reactions. The possibility of genetic variations introduced during repeated cycles of cell growth makes Mabs difficult to handle and potentially unreliable. Intact antibodies are known, due to their size, to remain in the circulation causing putative damage systematically or in non-targeted organs [16].

On the other hand, antibody fragments such as single-chain variable fragments (scFv) may be the smallest antigen-binding fragments, and they were originally developed to simplify the expression of antigen-binding fragments [17]–[19]. Also, scFv has been employed in several therapeutic models [20]–[23]. Dendritic cells (DCs) strongly upregulate the expression of costimulatory molecules and production of cytokines, and thus, they constitute the most potent antigen presenting cells (APCs), which are capable of stimulating naïve antigen-specific T cells and inducing a primary immune reaction. In addition, these cells have been used for therapy in several disease models [24]. Strategies that have been used to adapt this biological potential to therapy include manipulating DCs by co-culturing (pulsing) with whole antigens, defined antigenic peptides or total RNA, or genetically modifying DCs with genes encoding specific antigens. Among these strategies, genetically modifying DCs with genes encoding specific tumor antigens is a potential therapy. Therefore, in this study, we transfected DCs with scFv from Mab 7.B12 (Ab2-β). Our constructs had the typical structure of scFv, with the variable domain of the immunoglobulin heavy chain (VH) linked to the light chain (VL) via a flexible peptide linker in a VH-VL orientation [25]. We showed that DCs transfected with scFv were capable of efficiently activating the proliferation of total lymph node cells and inducing a decrease in lung infection. The engineering of scFv associated with DCs is a promising therapy in the experimental PCM model.

## Results

### Cloning and expression of Ab2-β Mab scFv in mammalian cells

**Figure 1:**
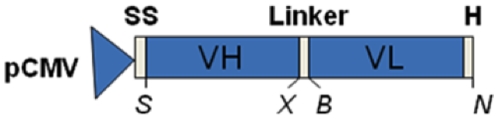
Design of scFv in pMAC/PS vector. The mammalian expression plasmid pMAC/PS drives the expression of scFv using the human CMV immediate early promoter. We show the schematic representation of the scFv expression cassette, highlighting the restriction sites added. The coding regions were: VH for heavy variable domain; VL for light variable domain; H for six histidine tags; SS for signal sequence; S for *Sma I*; X for *Xba I*; B for *Bgl II*; and N for *Nco I*.

Ab2-β Mab VH and VL domain gene sequences were determined and sub-cloned into the pIg16 vector [19] to enable the assembly of scFv. The scFv gene was then transferred to the pMAC/PS vector so that it could be expressed in mammalian cells. The expression cassette is shown in Figure 1.

**Figure 2:**
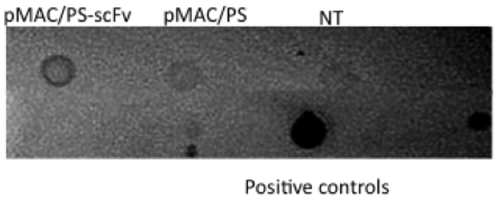
scFv production in transfected CHO cells. CHO cells were transfected with pMAC/PS (empty vector) or pMAC/PS-scFv and after 48 hours, the supernatants were collected, and the secretion of scFv was measured by dot immunoblotting. Supernatants from non-transfected CHO cell were used as the control (NT).

To assess its transient expression in mammalian cells, the scFv protein was measured in the culture supernatants of transfected CHO cells. After 48 hours, the supernatants were collected, and scFv secretion was measured by dot immunoblotting. We detected scFv in only supernatants from pMAC/PS-scFv-transfected CHO cells and not in supernatants from the controls (empty vector-transfected or non-transfected CHO cells) (Figure 2).

### scFv gene expression

**Figure 3:**
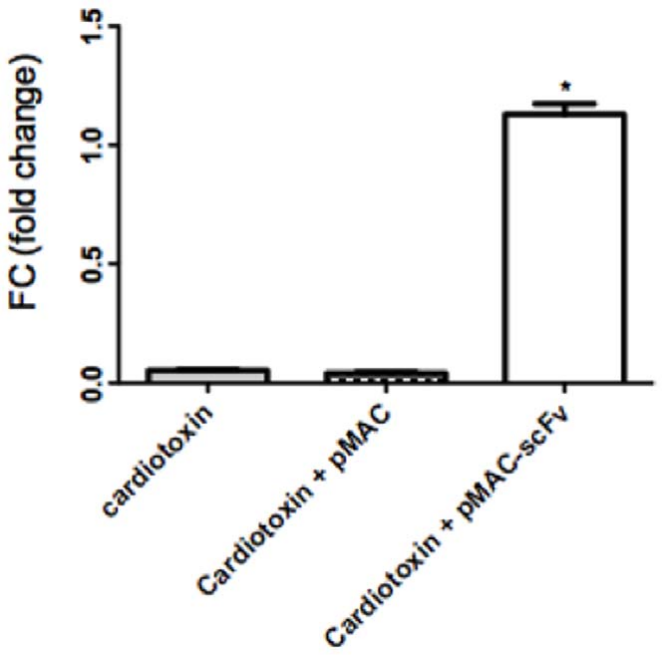
Real-time PCR. Total inguinal and popliteal lymph node cells were obtained after immunization with pMAC/PS-scFv. The mRNA was extracted, and the expression of the scFv gene was measured by real-time PCR using a specific primer for the VH counterpart of scFv. The fold change was calculated based on GAPDH mRNA. Controls without reverse transcriptase were used for each pair. *p<0.05 compared with the controls (cardiotoxin or cardiotoxin plus empty pMAC). Results are representative of 3 independent experiments in which 8 mice in each group were used.

DCs have the capacity to recognize, capture, process and present antigen, and to migrate to the lymph nodes and activate T lymphocytes through antigen presentation. To analyze the capacity of these cells to specifically respond to a DNA immunization through migration to the lymph nodes and expressing the Ab2-β Mab scFv, we intramuscularly immunized BALB/c mice with cardiotoxin, a potent adjuvant that induce a local inflammatory stimulus, five days before they received an intramuscular injection of the pMAC/PS-scFv or the empty vector. After 24 hours, we removed the inguinal and popliteal lymph nodes to verify the expression of the scFv gene. For this analysis, we extracted total RNA from the lymph nodes and measured the scFv expression by employing real-time PCR with specific primers for the VH counterpart of the scFv gene. The results showed that scFv gene expression increased only in animals that received pMAC/PS-scFv. In the controls, which received only the cardiotoxin stimulus or the empty vector, there was no increase in gene expression (Figure 3).

### DCs transfected with pMAC/PS-scFv stimulated lymph node cells *in vivo*

**Figure 4:**
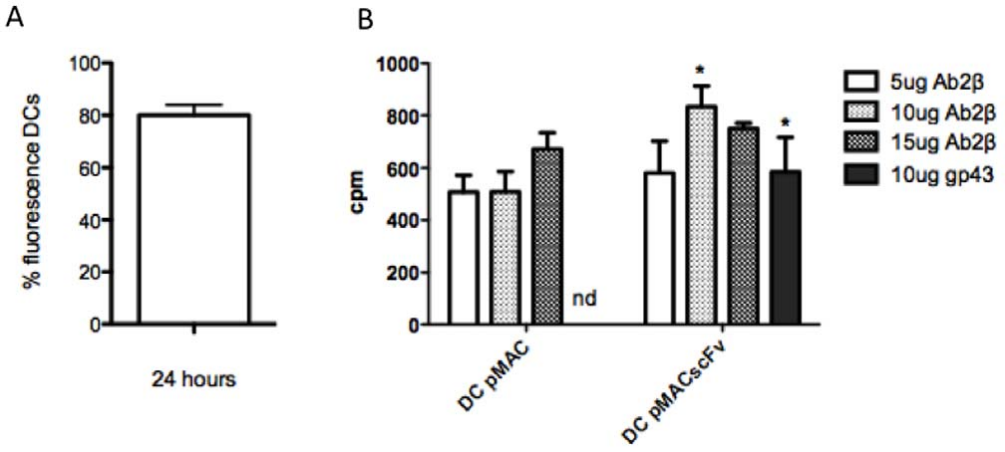
Proliferative response. (A) percentage of DCs transfected; (B) Proliferation of lymph node cells from immunized mice with DC-pMAC-scFv and re-stimulated *in vitro* with Ab2-β MAb (5, 10 and 15 µg/mL) or 10 µg of gp43. *p<0.05 compared with the control (DCs transfected with pMAC/PS). Results are representative of 3 independent experiments in which 6 mice in each group were used. nd – not detected.

To analyze the percentage of transfected DC, the cells were co-transfected with a GFP-Neo vector, and the result was determined by percentage of fluorescence cell. Our date showed that the transfection was approximately 80% successful (Figure 4 A). After that, to verify the capacity of DCs in presenting antigen and activating T cells, BALB/c mice were intramuscularly immunized with DCs transfected with pMAC/PS (control) or pMAC/PS-scFv. One week later, the popliteal and inguinal lymph nodes were collected and cultivated *in vitro* in the presence of different concentrations of Ab2-β Mab (5, 10 and 15 µg/mL) or 10 µg of gp43. The results showed that DCs transfected with pMAC/PS-scFv induced a significant proliferative response mainly in the presence of 10 µg/mL of the antibody. We also observed that re-stimulation *in vitro* with gp43 led to significant proliferation only in lymph node cells from mice that received pMAC/PS scFv immunization (Figure 4 B).

### Immunotherapy

**Figure 5:**
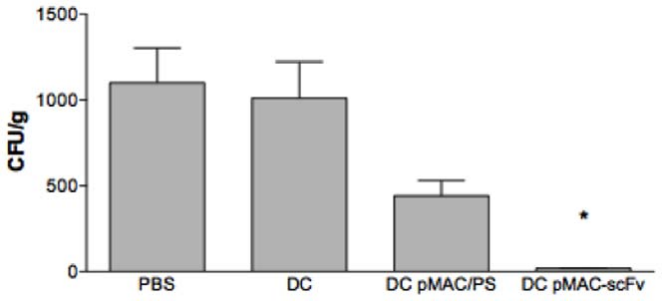
Protection against *Paracoccidioides brasiliensis* infection. Animals were intratracheally infected with the same amount of Pb18 virulent yeast. After 14 and 21 days, the experimental groups were treated with PBS, DCs, DCs transfected with the empty vector (DC pMAC) or DCs transfected with the scFv expression vector (DC pMAC-scFv). After one week, the mice were sacrificed, and the Pb18 CFUs of the lungs were determined. *p<0.05 compared with the controls (treated with PBS, DCs or DC pMAC/PS). Results are representative of 3 independent experiments in which 6 mice in each group were used.

To analyze whether DCs transfected with pMAC/PS-scFv were capable of inducing an efficient therapeutic effect, BALB/c mice were intratracheally infected with 1×10^6^ of Pb18 virulent yeast. After 14 and 21 days, they were treated intramuscularly with PBS, DCs, DC-pMAC/PS or DC-pMAC/PS-scFv. One week after the last treatment, the animals were sacrificed, and the lungs were collected for CFU analysis. The results showed a significant decrease in fungal growth only in animals treated with DCs transfected with pMAC/PS-scFv when we compared with the groups of animals that received PBS, DCs, or DCs transfected with pMAC/PS (Figure 5).

## Discussion

Infection with *P. brasiliensis* remains one of the main fungal infections in Latin America. Thus, the need to develop a novel immunotherapeutic model for this infection is important to public health. In this investigation, we engineered a scFv from the Ab2-β of the gp43 protein of *P. brasiliensis* and transfected it into DCs to investigate the capacity of these cells to efficiently induce a therapeutic effect in an experimental PCM model.

An important problem to be resolved in the PCM is the treatment. Frequently, several years of treatment are required and in the some case are not effective. Sulfonamides, ketaconazole, itraconazole, fluconazole and anphotericin B have been used in the treatment of PCM [26]. However, the treatment is long and hepatotoxic, then, the search for new alternatives for treating is necessary. Novel treatments may be found among new classes of drugs, or agents capable of modulating the immune response, such as scFv.

The scFv antibodies have been used in several immunotherapeutic experiments. In particular, KT-scFv (yeast killer toxin) exerted a therapeutic effect against *Candida albicans* rat vaginal infection [27], [28]. In serogroup B *Neisseria meningitides* (MenB), the immunization with scFv from capsular polysaccharide showed the potential of vaccination with genes encoding capsular mimics in providing protection against MenB [29]. Along these lines, we originally exploited the scFv derived from VH and VL domains of Ab2-β Mab as a functional internal image of gp43 from *P. brasiliensis*. Thus, we analyzed the antifungal activity when transfected into DCs, which are the most potent APCs identified thus far and are crucial for priming the immune response.

First, we analyzed whether a mammalian vector containing the scFv (pMAC/PS-scFv) could express the protein in eukaryotic cells. Therefore, we transfected the pMAC/PS-scFv into CHO cells, and after 24 hours, we measured the secretion of scFv by dot immunoblotting. Knowing that eukaryotic cells were able to express and secrete scFv *in vitro*, we intramuscularly immunized Balb/c mice with pMAC/PS-scFv, and after one day, we observed the presence of scFv mRNA in popliteal and inguinal lymph nodes.

Following the intramuscular injection of a plasmid DNA vaccine, most antigen expression occurs in transfected myocytes at the site of injection [30]. Accumulating evidence, however, suggests that immune priming is initiated by professional APCs rather than by these myocytes [31]–[33]. Because professional APCs are not typically found in normal muscle tissue, they presumably migrate to the site of DNA inoculation in response to inflammatory or chemotactic signals [34]–[36]. Infiltrating APCs may then either take up the plasmid DNA directly or cross-present the expressed antigen to initiate immune responses [37], [38]. DCs may be one of the APCs that migrate to the regional lymph nodes because among all of the presenting cells, DCs are mainly responsible for this process.

After observing that DNA-scFv immunization primed T cells in vivo, we analyzed whether DCs could also efficiently present scFv to all lymph node cells. For this experiment, we generated DCs from the bone marrow and transfected them with pMAC/PS-scFv. BALB/c mice were subcutaneously immunized twice with these transfected DCs. The results showed that lymph node cells, when re-stimulated *in vitro* with Ab2-β, were capable of inducing proliferation, and we observed that scFv mimicked the original gp43 antigen. According to several authors, DCs are one of the most important cells responsible for activating an efficient immune response, and these cells have been used as tumor treatments in immunotherapy models [39], [40].

DCs are an attractive target for therapeutic manipulation of the immune system to enhance an otherwise insufficient immune response to PCM. However, the complexity of the DC system requires their rational manipulation to achieve therapeutic immunity. Therefore, with scFv-transfected DCs, we reduced the lung infection of BALB/c mice infected previously with virulent Pb18 yeast. However, the mechanism of protection is unknown. An efficient cellular immune response with IFNγ-secreting CD4^+^ and CD8^+^ T cells is responsible for the decrease of *P. brasiliensis* in the organs during experimental infection. Some work has shown that transfected DCs could activate both CD4^+^ and CD8^+^ T cells. Shibagaki *et al.* used tumor-associated antigens containing the HIV TAT protein transduction domain (PTD) to transduce DCs [41], [42]. This fusion protein efficiently transduced DCs and was processed by proteasomes for MHC class I-dependent presentation to cytotoxic T lymphocytes. In addition, the TAT-containing antigen was also presented to CD4^+^ T cells as efficiently as native antigen. Finally, TAT-antigen-transduced DCs induced antigen-specific cytotoxic T lymphocytes *in vivo* through vaccination against tumors [41], [42]. Therefore, our results suggested an efficient T-cell activation may be induced by using transfected DCs.

Thus, we conclude that immunotherapy based on DCs transfected with scFv will become an essential component in PCM experiments to decrease infection and activate T cells.

## Materials and Methods

### Ethics Statement

The protocol was approved by the Committee on the Ethics of Animal Experiments of the Universidade de São Paulo in 02/2008 (Permit Number: 168).

### Mice

In the present study, we used 8- to 12-week-old female BALB/c mice obtained from the animal laboratory facility of the University of São Paulo.

### *Paracoccidioides brasiliensis* strains

The yeast form of the highly virulent *P. brasiliensis* strain 18 (Pb18) grown on Sabouraud agar (Neogen, Lansing-Michigan) was used for the infection assays.

### Preparation of fungal antigens

To purify gp43, the *P. brasiliensis* B-339 strain was prepared as described by Gesztesi et al [43] and passed through an adsorbent column consisting of murine anti-gp43 monoclonal antibody (Mab) 17C (IgG2a, k light chain) [44] coupled to an Affi-gel 10 column (Bio-Rad Laboratories, Hercules, CA, USA). The gp43 protein was eluted with 0.1 M citric acid buffer (pH 2.8), neutralized with 1 M Tris (pH 9.0), and further concentrated in a 10K Amicon apparatus (Amicon Division, Beverly, MA, USA). Protein content was determined using the Bradford method [45], and each purification step was monitored by sodium dodecyl sulfate-polyacrylamide gel electrophoresis (SDS-PAGE) [46].

### Production of Mab Ab2-β 7.B12

A hybridoma was produced as described [11]. Large amounts of Mab 7.B12 (IgG1, k light chain) were obtained by production of ascites in BALB/c mice previously primed with Pristaine (Sigma-Aldrich, St. Louis, USA). Mabs were purified from ascites fluids by affinity chromatography in a Protein G column (Amersham – Uppsala, Sweden). The antibodies were dialyzed against PBS and quantified.

### Enrichment of DCs

Bone marrow-derived DCs were generated according to described methods [47]. Femurs and tibias were flushed with 3–5 ml of PBS in 1% BSA. Bone marrow cells were allowed to differentiate into DCs through culturing in RPMI (Sigma-Aldrich, St. Louis, USA) supplemented with 10% Fetal Calf Serum (FCS) (Cultilab, Brasil), 10 mg/mL gentamicin (Schering-Plough, USA), and recombinant cytokine GM-CSF (50 ng/mL) (Peprotech, Brasil) for 7 days. On days 3 and 5, the nonadherent cells (granulocytes and lymphocytes) were removed, and media and growth factors were replaced. On day 7, the nonadherent cells were removed and analyzed by FACS using DC cell surface markers. More than 90% of the cells expressed CD11c^+^MHC classII^+^ (data not shown).

### Cloning and sequencing of 7.B12 variable regions

The hybridoma 7.B12 was grown in RPMI medium (Sigma-Aldrich, St. Louis, USA) containing 10% FCS (Cultilab, Brazil). Approximately 10^6^ cells were centrifuged and washed with PBS (150 mM NaCl, 10 mM Na_2_H_2_PO_4_, pH 7.2). Total RNA was isolated (TRIZOL, Invitrogen, Scotland, UK)), and the cDNAs that encode the VH and VL domains were synthesized using the SuperScript III Kit for RT-PCR (Invitrogen – Carlsbad, CA, USA), according to the manufacturer's instructions. For cDNA synthesis, the 1.25-µM primers used were Κ18 (5′TACAGTTGGTGCAGCATC3′) for CΚ and γ1 (5′TGGACAGGGATCCAGAGTTCCAGGTCACT3′) for Cγ [48]. For the amplification of the heavy and light chain V region, cDNAs were synthesized using a library of sense primers previously described [49], [50], and the same anti-sense primers were used for cDNA synthesis. The samples were subjected to 25 thermal cycles of 94°C for 1 min and 72°C for 1 min. Amplified VH and VL cDNAs were cloned into the pGEM-T easy vector (Promega, Madison, WI) following the manufacturer's instructions. Three clones of each variable region were sequenced in both directions. DNA sequencing reactions were performed using the DYEnamic ET Dye Terminator Cycle Sequencing (MegaBACE) Kit and run on the MegaBACE 500 Plus capillary sequencer.

### scFv construction

Based on the sequence determined, new oligonucleotides were designed and synthesized with the goal to clone the VH and VL domains into the mammalian expression vector pMAC/PS, a pMAC [51] that uses the cytomegalovirus IE1 promoter to drive the expression of the scFv fused to a immunoglobulin signal peptide. The primers were 5′*CCCGGG*TGAAGCTGGTGGAG3′ and 5′*TCTAGA*GGAGACGGTGACCGTGG3′ for VH and 5′*AGATCT*CCAAATTGTTCTCACCCAGTC3′ and 5′*CCATGG* TGATGATGGTGATGGTGTTTGATTTCCAGCTTGG3′ for VL (NCBI reference sequence: HQ326123 for VL and HQ322121 for VH). The generated amplicons harbored endonuclease sites (shown in italics) *Sma* I and *Xba* I for VH and *Bgl* II and *Nco* I for VL. Additionally, a sequence encoding a His tag was introduced in the VL 3′(underlined). The amplified domain genes were first cloned into the pIG 16 vector [19] for scFv assembly, using *SmaI* and *EcoRI* restriction enzymes, and then, they were transferred to the final vector pMAC/PS (http://www.unb.br/ib/cel/imol/vetor/pMACPS.html).

### scFv secretion

To analyze scFv production in mammalian cells, the protein was detected in the culture supernatant of transfected Chinese Hamster Ovarian (CHO) cells. The CHO cells were transfected (using lipofectamine, Invitrogen – Carlsbad, CA, USA) with an empty or scFv-carrying pMAC/PS vector. After 48 hours, the supernatants were collected, and the analysis was performed using dot immunoblotting. For this assay, 10 µL of the supernatants were adsorbed to a nitrocellulose membrane (Hybond-C, GE-Healthcare) and blocked with 1% BSA in PBS. After washes with PBST (0.05% Tween 20 in PBS), the membrane was incubated with a mouse anti-HIS antibody (1∶2,500) (Sigma-Aldrich, Carlsbad, CA, USA), followed by the addition of horseradish peroxidase (HRP)-conjugated rabbit anti-mouse IgG (Thermo-Scientific, 1∶5,000). The membrane was developed with diaminobenzidin (DABI) after the addition of mouse antibody anti-immunoglobulin (anti-IgG) and marked with peroxidase (1∶5000). As a positive control, we used *P. brasiliensis* (Ab1, 20 ug/mL).

### Proliferative response assay

Balb/c mice were immunized intramuscularly with DCs transfected (JetPEI Macrophage, Polyplus transfection) with pMAC/PS-scFv (1×10^6^ cells and 20 µg of vector with scFv). One week later, the mice were sacrificed, and the inguinal and popliteal lymph nodes were excised for cell isolation. For a control, we used DCs transfected with an empty vector. To calculate the percentage of transfected DC, the cells were co-transfected with a GFP-Neo vector, and after 24 hours the analysis was conducted using fluorescence microscopy. Lymph node cell suspensions in complete medium were plated at a concentration of 2×10^5^ cells per well in 96-well plates in the presence of Ab2-β (5, 10 and 15 µg/mL) or 10 µg of gp43. Cells were co-cultured for 72 h and then pulsed for 16 h with [^3^H]-labeled thymidine (1 µCi/mL) (Amershan, UK). Cells were collected with an automated cell harvester, and the incorporated radioactivity was measured by liquid scintillation spectrometry. The lymphoproliferation results were evaluated in accordance with the incorporation of [^3^H]-labeled thymidine (Amershan, UK). These results are presented as the mean counts per minute (CPM) (PerkinElmer).

### scFv gene expression in the lymph nodes

To analyze the capacity of DCs in presenting scFv in the inguinal and popliteal lymph nodes, we intramuscularly immunized BALB/c mice with 50 µL of the adjuvant cardiotoxin (7 µg/animal) [52] five days before we injected the pMAC/PS-scFv (10 µg/animal) or only the vector (10 µg/animal). After 24 hours, we obtained the total lymph node cells, and total RNA was extracted for real-time PCR using specific primers for the VH counterpart of scFv (forward – 5′GGGTCCGTGAAACTCTCCTG3′ and reverse – 5′CCCACTCCAGCCTCTTCTCT3′) domain of the antibody Ab2-β. As a control, we used specific primers for murine GAPDH (forward – 5′CCTGCACCACCAACTGCTTA3′ and reverse – 5′AGTGATGGCATGGACTGTGG3′) (NCBI Reference Sequence: NC_000072.5). For the real-time PCR reactions, we used the SYBR GREEN Kit (Invitrogen – Crlsbad, CA, USA) at concentrations recommended by the manufacturer. The analysis was conducted using the F7500 real-time PCR (Applied Biosystem). The fold change was calculated based on constitutive GAPDH mRNA levels. Controls without reverse transcriptase were also run for each pair.

### BALB/c therapy

Mice were challenged with an intratracheal inoculation of 1×10^6^ yeast cells of the virulent Pb 18 strain. After 21 and 28 days, the animals were treated in 4 differents groups and received by intramuscular injection therapeutic doses of: Group 1: only PBS (50 µL); Group 2: DCs (1×10^6^ cells); Group 3: DC-pMAC/PS (1×10^6^ cells transfected with 20 µg of vector) or Group 4: DCs -pMAC/PS-scFv (1×10^6^ cells transfected with 20 µg of vector containing scFv). After seven days, all animals were sacrificed, and the lungs were removed for colony forming unit (CFU) analysis.

### Statistics

Statistical comparisons were made by analysis of variance and the Tukey-Kramer test. The mean and standard error (SE) values are reported.
